# Tobacco Smoking: Patterns, Health Consequences for Adults, and the Long-term Health of the Offspring

**DOI:** 10.5539/gjhs.v4n4p62

**Published:** 2012-05-31

**Authors:** Gert S. Maritz, Muyunda Mutemwa

**Affiliations:** 1 Department of Medical Biosciences, University of the Western Cape, Bellville, South Africa

**Keywords:** smoking, nicotine, health, health of offspring, tobacco use, disease patterns, maternal smoking, nicotine replacement therapy

## Abstract

Tobacco use started several centuries ago and increased markedly after the invention of the cigarette making machine. Once people start smoking they find it difficult to quit the habit. This is due to the addictive effect of nicotine in tobacco smoke. Various epidemiologic and laboratory studies clearly showed that smoking is associated with various diseases such as heart diseases, asthma and emphysema and the associated increase in morbidity and mortality of smokers. Several studies implicate nicotine as the causative factor in tobacco smoke. Apart from nicotine, various carcinogens also occur in tobacco smoke resulting in an increase in the incidence of cancer in smokers. While the smoking habit is decreasing in developed countries, tobacco use increases in the developing countries. Smoking prevalence is also highest in poor communities and amongst those with low education levels. It is important to note that, although ther is a decline in the number of smokers in the developed countries, there is a three to four decades lag between the peak in smoking prevalence and the subsequent peak in smoking related mortality. It has been shown that maternal smoking induces respiratory diseases in the offspring. There is also evidence that parental smoking may program the offspring to develop certain diseases later in life. Various studies showed that maternal nicotine exposure during pregnancy and lactation via tobacco smoke of nicotine replacement therapy (NRT), program the offspring to develop compromised lung structure later in life with the consequent compromised lung function. This implies that NRT is not an option to assist pregnant or lactating smokers to quit the habit. Even paternal smoking may have an adverse effect on the health of the offspring since it has been shown that 2^nd^ and 3^rd^ hand smoking have adverse health consequences for those exposed to it.

## 1. Introduction

Tobacco is used since around the first century BC by Native Americans for ceremonial purposes. When the first Europeans arrived in America in the 15^th^ century it was already widely used. Following the the invention and development of the cigarette making machine to produce large quantities of cigarettes quickly, cigarettes were more readily available. Consequently the use of cigarettes increased markedly. During the 1990s approximately a billion people were daily smokers in the world. Of these around 47% were adult men and about 12% adult women (Foulds, 2008). Users of tobacco quickly become addicted to it due to the presence of nicotine in tobacco. Nicotine activates the same reward pathways in the brain than other drugs of abuse such as cocaine or amphetamines do, although to a lesser degree. Research has shown that nicotine increases the level of dopamine in the brain, resulting in feelings of pleasure and well-being (Benowitz & Jacob, 1984). However, evidence accumulated indicating that smoking and nicotine various respiratory ailments such as asthma and wheezing, cardiovascular disease and cancer. Epidemiological (Landau, 2008) and laboratory research (Maritz & Harding, 2011) furthermore indicate that maternal smoking is inducing diseases such as respiratory disease in the offspring.

Recent studies suggest that many of the diseases associated with tobacco smoke are actually induced by nicotine, the addictive substance in tobacco (Maritz & Windvogel, 2003; Petre et al., 2011; Sekhon, Keller, Benowitz, & Spindel, 2001). These diseases adversely impact on the productivity of individuals exposed to first, second, and third hand smoke in that it adversely impact on the health of such persons and increased hospital admissions.

The cost of smoking of smoking is estimated to be about $197 billion annualy. This includes health care costs of around $96 billion per year, as well as a loss in production due to absence from work due to illness in the region of $97 billion per annum (C.D.C., 2010). It is clear that tobacco smoking is adversely affecting health in general globally. This is illustrated by the fact that tobacco smoking results in more deaths worldwide than deaths caused by AIDS, legal and illegal drugs, road accidents, murder and suicide, combined (Ash research report, 2007). Tobacco associated deaths is already estimated to be approximately 5.4 million deaths annually (Ezzati & Lopez, 2003; W.H.O., 2008). It is projected thatif current smoking trends are maintained, deaths due to smoking may increase to more than 8 million per year by 2030. It is also estimated that around 80% of these deaths will be in the developing world due to an increase in tobacco consumption (C.D.C., 2008). According to reports the lifespan of tobacco users is reduced by about 13 to 14 years compared to that of non-smokers (CDC report, 2002). This figure does not take the effect of maternal smoking or maternal use of nicotine to quit smoking during pregnancy on the health and lifespan of the offspring in the longer term into consideration

The objective of this review is therefore, to: 1) record the factors determining patterns of smoke exposure by smokers and non-smokers, 2) report on the consequences of maternal exposure to tobacco smoke and/or nicotine on the health of the offspring, especially in the longer term, and 3) discuss the appropriateness of NRT to assist especially pregnant and lactating mothers to quit the habit.

## 2. Types of Tobacco Smoke Exposure

### 2.1 First Hand Smoke Exposure

First hand smoke is the smoke inhaled by a smoker. It has been shown that tobacco use is one of the major preventable couses of death and disability in the United States (US). About 440,000 smoke related deaths occur every year. This implies that aboutr one in five of all deaths are attributable to tobacco use. Worldwide it is estimated to be 5.4 million/year and increasing (Ezzati& Lopez, 2003; W.H.O., 2008).

### 2.2 Second Hand Smoke Exposure

This is tobacco smoke inhaled by non-smokers. It is also referred to as environmental tobacco smoke. There are 2 kinds of second hand smoke, namely 1) side stream smoke which is released by the burning end of a cigarette, or cigar or pipe and, 2) main stream smoke which is exhaled by a smoker. According to the surgeon general’s report of 2006, more than 126 million people are exposed to second hand smoke. This resulted in 50 000 deaths in the USA, which means that the worldwide figure will be much higher. This means that to be in the presence of a smoker will also be harmful for non-smokers. The seriousness of smoking in public places is emphasized by the fact that there is no safe level of second hand smoke (Spiegler, 2011).

Apart from tobacco smoke in public places, individuals in cars driven by smokers are exposed to extremely elevated concentrations of particulate matter. The concentrations of these smoke related particulate matter is 60 times greater in unventilated cars than in a smoke free home, and up to 27 times greater than in a smoker’s home. Even under full ventilation, the particulate matter in the home of a smoker is at least 13 times that of outdoor concentrations (Ott, Klepeis, & Switzer, 2007). Especially children are compromised by the exposure to these high levels of particulate matter since they are still growing and therefore more susceptible to the adverse effects of tobacco smoke particles on their health in the longer term.

There are more than 50 sunstances in 2nd that are carcinogenic. For example, exposure to 2nd hand smoke induces cancer of the lungs and nasal sinuses. It also induces infection in the respiratory tract as well as heart disease. Since there is no safe amount of second hand smoke, children, pregnant women, older persons, as well as people with heart or respiratory problems should be particularly careful (Okoli, Kelly, & Hahn, 2007). This is of particular importance since it has been shown that the blood nicotine levels of people exposed to 2^nd^ hand smoke resembles that of smokers, and that 2nd hand smoking is responsible for up to 3000 lung cancer deaths in the USA (USEP, 1992). It is also associated with diseases of the cardiovascular and respiratory systems. This shows that 2^nd^ hand smoke is a formidable health risk to non-smokers.

Studies showed that children exposed to parental smoke are more likely to smoke when reaching their teenage years. They will also find it harder to quit in adulthood. Such associations suggest that second-hand smoke acts on the brain of teenagers to promote smoking behaviour even in adulthood (Brody et al., 2011). Furthermore, chronic exposure to tobacco smoke could result in even higher brain nicotine levels, which may explain why second hand smoke exposure increases vulnerability to nicotine addiction (Brody et al., 2011).

### 2.3 Third Hand Tobacco Exposure

This is cigarette smoke deposits on furniture, clothing and other surfaces. It includes invisible smoke left in the air after a cigarette is extinguished (Winickoff et al., 2009). It is actually the residue of second hand smoke. It has been illustrated that that the residue of nicotine that occurs on the surfaces of, for example furniture, can react with other chemicals in the air to form carcinogens. The nicotine that is released from a burning cigarette in the form of vapour adsorbs strongly onto indoor surfaces such as walls, carpets, furniture, drapes and loose household dust. In addition to the proven adverse effects of first and second hand smoking on health, third hand smoke may also pose a threat to especially babies and toddlers (Tuma, 2010; Winickoff et al., 2009).

Recent studies illustrated that high levels of tobacco toxins remain well beyond the period of active smoking. These toxins are in the form of particulate matter that is deposited onto every surface in the house. These volatile toxic compounds evaporated into the air over an extended period of time and in this way result in a continuous exposure of the inhabitants to these toxins (General, 2006).

## 3. Patterns of Tobacco Use

### 3.1 Developed Countries

During 1965, 42% of adult American smoked. This figure dropped by more than 50% to 20.6% in 2009. However, this figure remained unchanged since 2005. This is in contrast to the marked decreases of past decades. Also, not al smoker smoke every day. An estimated 78% of smokers smoke every day, while 22% smoke less frequently than daily (C.D.C., 2010).

Apart from the above the pattern of tobacco use in the US and elsewhere, it also differs among socio-demographic groups. In the 1960s the prevalence of cigarette smoking was much higher in males than in females because over 50% of males were smokers as oppose to the 25% of female smokers. Since then the male/female differences in smoking prevalence decreased to 23.5% of men and 17.9% of women (C.D.C., 2010). There are large differences in the smoking prevalence of males and females across countries. For example, in India there are aproximately 12 times more male smokers than female smokers, and in Japan and Pakistan thre are male smokers than female smokers. Contrary to the gender differences in the above countries, there is almost no male/female differences in the European countries and the US (W.H.O., 2005). In many developed countries the gender gap between male and female smokers recently narrowed drastically mainly due to a sharp decline in smoking prevalence among the males (W.H.O., 2005) (C.D.C., 2010). This can probably be explained by the model suggested by lopez et al (1994) which indicate that the male/female prevalence of tobacco use worldwide can be divided into four stages, namely: Stage 1: where smoking prevalence in men increase first, and is followed 10 to 20 years later by a small increase in smoking prevalence in females. Stage 2: The prevalence in both males and females continue to increase, with the prevalence ratein males slightly higher than in females. Stage 3: The prevalence rate in males plateaus and then decrease to levels that compares with rates in females. During this stage female smoking prevalence increased, followed by a decrease but not as pronounced as in males. Stage 4: Smoking prevalence continues to decrease in both males and females until they are nearly equal.

It is interesting to note that in countries in Northern Europe and North America as well as Australia, it is those in disadvantaged circumstances socio-economically that are most likely to smoke cigarettes (Nicolaides-Bouman, 1993). It is also unlikely that the smokers in a low socio-economic situation will use nicotine replacement strategies or other strategies to quit smoking. This means that the impact of smoking on health in these communities will in all likelihood remain high. Apart from being unable to also afford any of these strategies, lower socioeconomic status of individuals are associated with lower awareness of the harms of smoking and misunderstanding around the harmful effects of nicotine. There is a need to improve knowledge of the dangers of smoking among the disadvantaged segments of the population in order to improve health and lower the mortality rate (Siahpush, McNeill, Hammond, & Fong, 2006). Smoking among women is linked to social disadvantage, with the highest rates among white women in working class households. Smoking appeared to provide a way of dealing with the demands of full-time caring of their families, as well as with the continuous effort to making ends meet (Hilary, 1987).

It has also been shown that the level of education plays an important role in that men and women of higher education levels smoke less than those of a low level of education. This emphasizes the importance of education in reducing the use of tobacco smoke worldwide and the consequent improvement of health in general.

Race differences are also apparent in that white men smoke less than black men. On the other hand, black females smoke less than their white counterparts (Kandel and Chen, 2000).

Studies showed that from the late 1990s women now outlive men in all countries of the world. Deaths associated with smoking accounted for about 40% - 60% of this gender gap (McCartney, Mahmood, Leyland, Batty, & Hunt, 2011), and is a reflection of the male/female differences in taking up smoking during the earlier decades (Lopez, Collishaw, & Piha, 1994). However, since the gap between the number of male and female smokers becomes smaller (W.H.O., 2005), it can be expected that the prevalence of smoking related diseases and deaths will increase amongst female smokers over time and thus reduce the gender gap.

According to the data from the Global Youth Tobacco Survey, the differences in smoking rates among boys and girls are less than the rates among adult males and females. Comparing smoking rates between boys and girls between the ages of 13 and 15 years, shows that the boys smoke only 2 to 3 times more than girls (Warren et al, 2006).

### 3.2 Developing Countries

Presently, tobacco use is the single largest and most preventable cause of premature adult death throughout the world. Unfortunately, the global tobacco epidemic shows little sign of abatement, primarily because of the rapid increase in tobacco use in developing countries. About 73% of the world’s smokers are in developing countries. According to estimates by the WHO about 50% of men and about 8% of women in developing countries are smokers. This is much higher than in developed countries. This can partly be attributed to a lower level of education and poverty of a large part of the population in these countries as well as the targeting of these countries for marketing of tobacco products by tobacco companies (Barry, 1991). If second hand and third hand smoking is taken into consideration, it is conceivable that almost the entire population in these countries are exposed to tobacco smoke. It is therefore, not surprising that the estimated number of deaths in these countries will increase from approximately one million in the 1990s to seven million in the 2020s or 2030s (Collishaw & Lopez, 1995).

## 4. Disease Patterns Related to Tobacco Smoke

Smoking is associated with various diseases of the cardiovascular and respiratory systems as well as cancer. It is also generally accepted that smoking reduces the average lifespan of smokers. It is estimated that, if current smoking tobacco consumption patterns are maintained, about 450 million adults will die as a consequence of tobacco smoke-related diseases between 2000 and 2050. Of the 450 million, about 50% will be between 30 and 69 years of age (Jha, 2009). In addition, second and third hand smoke contribute to the increase in smoke related morbidity and mortality.

It is disturbing that a major factor affecting public awareness of the health hazards of tobacco smoking is the three- to four-decade lag between the peak in smoking prevalence and the subsequent peak in smoking related mortality (Lopez et al., 1994). This means that when smoking prevalence decreases there will be an increase in smoking associated diseases because current mortality is most closely related to previous, not current levels of cigarette consumption (Winickoff et al., 2009). This essentially means that even when smoking declines dramatically in both developed and developing countries, the adverse effects thereof will be seen in the future. And since the effects of smoking and nicotine are heritable, the health consequences of parental and grand parental smoking will be evident only in generations to come. It might be worse in certain developing countries where poverty prevails.

Furhermore, the impact of smoking on the future health of the unborn baby is not generally explained in various journals and health reports. It has been shown that respiratory diseases in the offspring, such as asthma are higher when the parents smoke. However, the child might be born normal and only develop smoke related diseases later in life. In addition, the capacity of the smoke exposed offspring to protect itself against challenges from the environment, such as pollution and food additives may be less than in that of offspring of non-smoking parents. The level of health care, availability of food, level of education, level of income, and lifestyle all impact on the disease patterns in various regions and the offspring. If this is taken into account it will certainly further increase the morbidity and mortality rate globally.

## 5. Current Mortality and Disability from Smoking

Recent updates of indirect estimates of global tobacco mortality suggest that in 2000, 5.0 million deaths were caused by tobacco. About 2.6 million of those deaths occurred in low-income countries. Of these premature deaths, 3.7 million, or 72% were males. About 60 percent of male and 40 percent of female tobacco deaths were of persons between the ages 35 to 69 (Ezzati & Lopez, 2003).

In high-income countries and former socialist economies, the 1 million middle-aged male tobacco deaths were mostly due to heart disease (0.45 million) and lung cancer (0.21 million). In the other hand, in low-income countries, the major causes of death among the 1.3 million male tobacco-related deaths were cardiovascular disease (0.4 million), chronic obstructive pulmonary disease (0.2 million), other respiratory disease (chiefly tuberculosis, 0.2 million), and lung cancer (0.18 million). An example is that whilst the association between smoking and tuberculosis is so low that it is seldomly studied in high income countries such as the United Kingdom, it has been reported that smoking accounts for almost 40% of the tuberculosis deaths amongst middle-aged men in India (Jha, 2008 and 2009). This might partly be attributed to inadequate health services in low income countries.

## 6. Consequences of Parental Smoking on the Offspring in the Long Term

### 6.1 Behavior Patterns of the Offspring

There is evidence which suggests that maternal smoking during pregnancy is not associated with an increased likelihood that her children will also late attempt to smoke or become regular smokers. However, maternal smoking greatly increases the likelihood that if her children are exposed to smoke, such as 2^nd^ hand smoke in the house, they will become addicted to tobacco. This can be attributed to the nicotine in 2^nd^ hand smoke and the programming of the children to be more susceptible to nicotine addiction (Buka et al, 2003). This means that maternal smoking and thus the exposure of the fetus to nicotine during pregnancy may make the offspring more susceptible to the addictive effect of smoking during adolescence and adulthood (Buka, Shenassa, & Niaura, 2003). According to them the most likely explanation is that the toxins, such as nicotine, in tobacco smoke cross the placental barrier and change the genetic or epigenetic make up of the cells that is associated with the control of cell differentiation, and in this way program the cells to become more responsive to the effects of nicotine. This change is in all likelihood permanent. They further reported that maternal smoking is not increasing the likelihood of the offspring to become addicted to marijuana. This means that the mechanism that increases the likelihood of becoming addicted to nicotine is different from that of individuals that become addicted to marijuana.

In addition, maternal smoking during pregnancy is also associated with increased criminal and substance abuse outcomes in male and female offspring in adulthood (Brennan, Grekin, Mortensen, & Mednick, 2002). It is therefore evident that maternal smoking, and in all likelihood 2^nd^ hand smoke during the very early phases of the development of the fetus may increase the chances of the offspring to be more prone to diseasae and behavioural problems in adult life.

### 6.2 Maternal Smoking and Disease in the Offspring

Barker (2001) that changes in the environment within which the fetus and newborn develops may increase the risk of the offspring to develop chronic diseases later in life This can be attributed to developmental plasticity which enables the organism to adjust structure and function in response to the changing environment; the latter can be the external environment and/or in utero environment. The response to the environment occurs during critical windows of time during development and then become irreversible due to changes in the program that control structure, function and maintenance of the organism. Such plasticity allows for a range of phenotypes to develop from a single genotype in response to changes in the environment within which the fetus develops (Gluckman & Hanson, 2004). Maternal and paternal smoking is contributing to a change in the external environment as well as to the in utero environment and therefore, affects the fetal development during phases of high tissue plasticity. This may severely increase the vulnerability of the offspring to disease in adulthood (Gluckman, Hanson, & Mitchell, 2010).

There is ample evidence that maternal smoking during pregnancy and thus the exposure of the developing fetus to tobacco smoke *in utero*, adversely affects lung function of the offspring after birth. These functional deficits persist throughout childhood and also adult life (Landau, 2008; Sherrill, Lebowitz, Knudson, & Burrows, 1991). The adverse effects of tobacco smoke on the developing fetus and in particular the developing lung can partly be attributed to many of the around 4000 chemicals present in tobacco smoke. More and more evidence shows that smoking induces genetic polymorphisms with the related adverse impact on the structure and function of the respiratory system. The effect might not me immediately apparent but only after the individual is exposed to environmental toxins (Landau, 2008). This is conceivably due to a reduced capacity of the body to protect itself against the impact of environmental stressors.

Tobacco smoking and exposure to second-hand smoke are widely accepted to be causative factors for childhood asthma and chronic obstructive pulmonary disease (COPD) affecting nearly 3 billion people worldwide, predominantly in China, India, and Africa (The Lancet, 2007). Chronic obstructive pulmonary disease is irreversible and difficult to treat. Recent studies suggest that if the mother smokes during pregnancy, the baby’s susceptibility to childhood asthma and possibly COPD later in adulthood increased. This can be attributed to programming during a stage of high developmental plasticity (Bateson et al., 2004) and in this way increase the susceptibility of the lungs for respiratory disease. Although some diseases that are associated with tobacco smoking are thought to be caused by nicotine, some suggests that there is a lack of direct experimental evidence linking nicotine to COPD (Britton & Edwards, 2008). However, several studies clearly demonstrate that nicotine is directly and specifically responsible for inducing differentiation of developing lung cells from the normal to abnormal phenotypes (Maritz & Harding, 2011; Maritz, Mutemwa, & Kayigire, 2011; Rehan et al., 2007; Rehan et al., 2005; Rehan et al., 2007).

It is reported that more than 400,000 infants are exposed to maternal smoke per annum in the US alone. It can therefore be assumed that maternal smoking is a huge worldwide public health problem and the increased numbers of individuals taking up the smoking habit in developing countries will greatly contribute to this. Furthermore, given that the cost of advertising for smoking increased by over one billion dollars from 2001 to 2002, it is unlikely that the problem of smoking during pregnancy will go away within the near future. More direct and reliable information regarding the effect of smoking and nicotine on the health and social behavior of the offspring will have to be collected for educational purposes and to counter marketing strategies. It is, therefore, important to evaluate the organ-specific mechanisms whereby *in utero* smoke exposure affect organ structure and function before there is any real chance of developing strategies to prevent the adverse effects of smoking (Rehan, Asotra, & Torday, 2009).

The gaseous and soluble phases of cigarette smoke are sourcesof oxidants that contribute to the pathogenesis of chronic obstructivepulmonary disease (COPD). Fortunately, the respiratory tract has developed effective adaptive cellular mechanisms to limit oxidant damage. Numerous antioxidant enzymes and glutathione-dependent detoxification systems are increased in healthy smokers (Cantin, 2010). However, long term regular exposure to these oxidants, especially when a heavy smoker is also exposed to other sources of oxidants, such as air pollution and food sources, or when the antioxidant intake via the diet is inadequate, the oxidants may override the protection mechanisms of the respiratory system and in this way induce COPD over time. It is suggested that protection afforded by nutrients or antioxidants counterbalances the injury imposed by environmental agents (Thomas, 2005). Individuals with a compromised capacity to protect themselves against environmental stressors will be much more susceptible to diseases such as COPD, cardiovascular disease and cancer. Due to the lower capacity to protect them against disease means that they are more dependent on external sources for antioxidants for protection. Therefore, poor populations are expected to more readily develop diseases due to inadequate internal and external protection against smoking related diseases.

It has been proposed that smoking induces premature aging with the concomitant increased susceptibility to disease. This tobacco smoke induced premature aging is linked to an antioxidant/oxidant imbalance in the adult as well as offspring of smoking parents. This is supported by the fact that cigarette smoke contains 10^17^ oxidant molecules per puff. The oxidants in cigarette smoke cause lung injury by a number of mechanisms including the depletion of glutathione and other antioxidants, the initiation of redox cycling mechanisms, enhancement of the respiratory burst in neutrophils and macrophages, inactivation of protease inhibitors such as α1-antitrypsin inhibitor, and direct damage to lipids, nucleic acids and proteins. In addition, it has been shown that oxidative damage to mitochondrial DNA may play a significant role in normal aging (Lin & Flint Beal, 2003).

This hypothesis of oxidative stress and mitochondrial dysfunction remains one of themost attractive hypotheses of aging (Balaban, Nemoto, & Finkel, 2005). Studies by Nyunoya et al. (2006) showed, for example, that a single exposure to cigarette smoke inhibits normal fibroblast proliferation which is essential for lung repair and maintenance. Furthermore, multiple exposures to cigarette smoke induce irreversible senescence of these cells and consequently slower proliferation (Nyunoya et al., 2006) and thus impaired repair mechanisms. Oxidative damage is a major factor in the loss of physiological functions that occur in degenerative diseases and aging (Huang & Manton, 2004). This implies that this stress-induced premature senescence renders the lungs more susceptible to damage by environmental oxidants and onset of COPD.

Unlike the adult lung, the fetal lung is unable to protect itself against inhaled foreign substances. This is because the expression of various pulmonary antioxidants is developmentally regulated in many species so that the expression is increased toward term gestation, as if in anticipation of birth into an oxygen-rich extra-uterine environment. Therefore, the lungs of prematurely born infants may be ill-adapted for protection against ROS (Asikainen & White, 2004; Valachovicova, Krajcovicova-Kudlackova, Ginter, & Paukova, 2003). The developing fetus is largely dependent on the mother for protection against oxidants. Because cigarette smoke is high in oxidants it drastically depletes plasma and tissue antioxidants of the mother (Fraga, Motchnik, Wyrobek, Rempel, & Ames, 1996), even after smoking a single cigarette (Tsuchiya et al., 2002). Consequently the protection afforded to the developing fetus and fetal respiratory system by the mother is compromised. It means that the fetus of the smoking mother, or the mother exposed to passive smoking, is much more vulnerable to the effects of oxidants from tobacco smoke which can result in epigenetic and DNA changes and thus a change in the program that controls the development of the respiratory system. This is of particular importance this may reduce the ability of the respiratory and other systems to protect itself against and an increase in the potential for disease even if the offspring don’t smoke themselves.

Furthermore, in addition to inducing overproduction of oxidants, nicotine exposure results in a decrease in the activity of superoxide dismutase (SOD) and catalase. It also results in a decrease in the levels of low molecular weight antioxidants such as vitamins C and E(Zaken, Kohen, & Ornoy, 2001). Along with the decrease in the antioxidant capacity of the body, concentrations of malondialdehyde (MDA) are increased, indicating oxidant damage to the cells (Halima, Khlifi, Rtibi, Elfazaa, & Gharbi, 2009; Özokutan et al., 2005). The increase in reactive oxygen species (ROS) levels, together with a decrease in the activities of enzymes with antioxidant function, results in an imbalance in the oxidant/antioxidant capacity. This imbalance is maintained long after nicotine withdrawal and becomes worse with age (Bruin et al., 2008). This means that the offspring of smokers and those using nicotine to quit smoking will be more prone to develop oxidative stress related diseases. In addition, oxidative stress also induce fetal programming which also contribute to the development of adult onset diseases during fetal life (Luo et al., 2006).

## 7. Maternal Nicotine Exposure and Health of the Offspring

Many of the adverse effects of smoking can be attributed to nicotine in first, second and third hand smoke (Maritz et al., 2011; Petre et al., 2011). Several laboratory studies showed that nicotine indeed induce changes in metabolism (Bruin et al., 2008; Maritz et al., 2011) and structure of organs (Holloway et al., 2005; Maritz et al., 2011; Sekhon et al., 2001). This is followed by a down regulation of organ function together with an increase in susceptibility to disease (Maritz & Windvogel, 2003).

### 7.1 Smoking and Nicotine and the Placenta

It has been suggested by Andres and day (2000) that maternal smoking during pregnancy may induce growth restriction in utero (IUGR). Since the risk of IUGR is significantly higher in women who smoke cigarettes with a higher nicotine content (Bainbridge & Smith, 2006), it is plausible that nicotine is the causative factor. It is therefore conceivable that placental perfusion during pregnancy will be adversely affected by maternal smoking as a result of nicotine-induced vasoconstriction in the placenta (Economides & Braithwaite, 1994). Contrary to this, it was shown by Bainbridge and Smith (2006) that nicotine is not affecting the feto-placental blood supply. It is therefore, unlikely that the nutrient supply to the developing fetus will be compromised by nicotine. This is supported by research that showed that exposure of pregnant rats to nicotine had no effect on the body weights of the offspring (Maritz & Windvogel, 2003).

It is therefore plausible that any contribution to IUGR in smokers is not at the level of the feto-placental perfusion but also at the level of placental function and/or uterine function (Maritz & Windvogel, 2003). This is supported by the fact that maternal smoking changes important aspects of placental function, such as progesterone production (Piasek, Blanuša, Kostial, & Laskey, 2001), estrogen metabolism(Zhu et al., 2002), amino acid transport (Pastrakuljic, Derewlany, & Koren, 1999), and the location and activity of drug metabolizing enzymes (Zdravkovic, Genbacev, McMaster, & Fisher, 2005). These changes may, conceivably, have a detrimental effect on fetal growth and development and contribute to IUGR in smokers.

In addition, smoking as well as nicotine also changes the placental structure which again will affect placental function. The type of structural alteration, and thus functional change, is likely to depend on the phase of pregnancy during which the placenta is exposed to smoke or nicotine (Genbacev, Bass, Joslin, & Fisher, 1995). The cumulative effects of changes in placental structural and functional may have an adverse effect on fetal growth and development and thus IUGR. The severity of the IUGR is determined by the level of smoke or nicotine exposure (Zdravkovic et al., 2005). A consequence of this insult is the induction of bronchopulmonary dysplasia which is associated with compromised lung function later in the life of the offspring (Barker et al., 1991).

### 7.2 Nicotine and the Lung

Nicotine acts on nicotine acetylcholine receptors (nAChRs) to mimic the effects of acetylcholine. High affinity nAChRs are found in the membranes of normal lung cells and in lung cancer cells of all histological types (Maneckjee & Minna, 1994; Maus et al., 1998; Pontieri, Tanda, Orzi, & Chiara, 1996). After the binding of nicotine to these receptors on the bronchial epithelial cells of rodents, proliferation of these cells commences. Nicotine also activate several pro-survival signals(Cattaneo, D’Atri, & Vicentini, 1997; Minna, 2003) such as increasing the activity of protein kinase C (PKC) in various human and murine lung cancer cell lines and Raf-1 (Chu, Guo, & Chen, 2005). The activation of these kinases result in theosphorylation of Bcl-2 which antagonize opioids-induced apoptotic signalling in lung cancer cells (Heeschen et al., 2001; Macklin, Maus, Pereira, Albuquerque, & Conti-Fine, 1998; Maneckjee & Minna, 1994). Nicotine also increases the phosphorylation of Akt in the lungs of nicotine-treated mice. It also activates Akt in human lung cancer cells.derived from smokers and is associated with tobacco-related carcinogenesis in the lung. These data suggest that while the activation of these kinases was observed in cultured cells transiently exposed to nicotine, nicotine directly or indirectly promotes lung carcinogenesis (Cattaneo et al., 1997; Maneckjee & Minna, 1994; West et al., 2003).

Furthermore, research by Guo, Chu, Abbeyquaye, & Chen (2005), Hartwell & Kastan (1994), and Vogelstein & Kinzler (1992) showed that long-term nicotine exposure results increases the susceptibility genetic instability. Gene amplification is a characteristic of gene instability. The latter requires 2 critical elements, namely an inappropriate cell cycle progression, and DNA damage. Long-term nicotine exposure, through the activation of Ras pathways and up regulating cyclin D1, disrupts the G1 arrest. It also enhances the production of ROS which alsoinduces DNA damage. This implies that exposure to nicotine via tobacco smoking or via NRT to quit smoking will make the lungs more prone to the development of cancer (Guo et al., 2005).

## 8. Nicotine Replacement Therapy

Various strategies are proposed to assist smokers to quit. These include prescription drugs such as bupropion (Roddy, 2004) and varenicline (Klesges et al., 2006). NRT is also an option to assist smoker with quitting the habit (Moore et al., 2009). This can be combined with joining support groups or to make use of individual counseling (Fiore et al., 2000). Also, NIDA-funded research illustrates that methoxsalen partially blocks the breakdown of nicotine and thereby maintain arelatively high nicotine content in the plasma and body tissues of the user. In this way the effectiveness of NRT suppress the urge to smoke for a longer period of time (Sellers et al., 2000) and thereby reduce the number of cigarettes smoked per day by an individual. The use of bupropion and varenicline is associated with various side effects. Suicidal thoughts are associated with bupropion and varenicline (Moore et al., 2011). It is therefore important that, although clinical data show that varenicline is effective and in general well tolerated by smokers, it must be further tested to assess its effect on smokers with a co-morbid condition (Garrison & Dugan, 2009). While there is a body of data available regarding the effects of nicotine on the future health of the offspring, no such information is available for the drugs such as bupropion.

NRT is a strategy proposed by various researchers and health professionals to assist smokers to quit the habit. Various nicotine containing products are currently available to provide nicotine in order to reduce the craving for smoking; these include nicotine containing gums, patches, lozenges, sprays (John Britton & Richard Edwards, 2008) as well as electronic cigarettes. NRT has been recommended to assist women to quit smoking when they become pregnant. In a position paper of the Ontario Medical Association (O.M.A) it was stated that it is a myth that pregnant women should never use nicotine replacement therapy. Despite the fact that in trying to explain why it is a myth, it is mentioned that nicotine, 1) through its reduction in uterine blood flow may contribute to spontaneous abortion, premature birth, low birth weight, perinatal mortality, and 2) that there is no safe dose of nicotine, especially since the fetus responds most adversely to nicotine administration (O.M.A, 1999). However, several studies clearly show that nicotine is not safe. Consequently it was suggested that all pregnant women, and those who plan a family, should stop smoking immediately because if a mother abstains from smoking during the first 3 months of her pregnancy, the risks to the fetus are the same as those of a fetus of a non-smoking mother (Ruiz, 2006). However, latest research on rats showed that nicotine adversely affects lung development even when the mother was only exposed to it during lactation (Maritz and Windvogel, 2003). This suggests that no smoking and no nicotine should be allowed during pregnancy and lactation. Therefore, despite the fact that NRT is widely prescribed an aid to stop smoking, it is questionable whether nicotine intake during pregnancy and lactation is safe for the fetus and neonate.

This is supported by the evidence derived from several studies that show that nicotine can damage the fetal lungs, heart, and the central nervous system. Nicotine is genotoxic (Kleinsasser et al., 2005) and its toxic effects persist in the fetus after administration has stopped (Ruiz, 2006). Studies in non-human primates clearly show that nicotine exposure during pregnancy increases the development of α_7_ nicotinic receptors in cells implicated in lung development. Pulmonary hypoplasia and other abnormalities in pulmonary and bronchial development have been found in the offspring after exposure to nicotine during gestation (Ezzati & Lopez, 2003; Maritz, 1997). It is also evident that nicotine exposure during development suppresses lysyl oxidase activity and this could contribute to the gradual deterioration of the lung parenchyma of the offspring (Maritz, 2008). Furthermore, nicotine induces peroxidation of membrane lipids (Mishra et al., 2008) which compromises the oxidant/anti-oxidant status of the lungs of the offspring. Nicotine also exposure *in vivo* decrease in the vitamin C and E content of the lungs of the offspring (Mishra et al., 2008) as well as decreasing the levels of the enzymes that catalyze the removal of antioxidants from the lung. This creates an oxidant/antioxidant imbalance which can cause changes in the program that controls homeostasis and lung aging in the offspring. This change in the program makes them more susceptible to respiratory diseases later in life (Maritz, 2009). This implies that, apart from its immediate effect in the lungs of those who use NRT, maternal nicotine intake during pregnancy and lactation will have a long-term effect on the maintenance of lung integrity and respiratory health of exposed offspring (Wallace, 2005).

## 9. Key Messages for the Individual Smoker

More than 50 years of epidemiology on smoking-related diseases have led to three key messages for individual adult smokers worldwide (Doll, Peto, Boreham, & Sutherland, 2004).

1. The eventual risk of death from smoking is high, with about one-half to two-thirds of long-term smokers eventually being killed by their addiction.
2. These deaths involve a substantial number of life years forgone. About half of all tobacco deaths occur at ages 35 to 69, resulting in the loss of about 20 to 25 years of life, compared with the life expectancy of nonsmokers.
3. Cessation works: those adults who quit before middle age avoid almost all the excess hazards of continued smoking.

Worldwide, about 80 percent of deaths among the 2.7 billion adults over age 30 involve vascular, respiratory, or neoplastic disease. Smoking is associated with an increase in the frequency of many of these diseases, although important differences exist between and across populations.

Parental smoking and nicotine replacement therapy should be stopped because it program the offspring to:

4. Have reduced resistance against stressors in the environment
5. Increase risk of diseases later in life such as respiratory disease, cancer, cardiovascular disease, and
6. Nicotine replacement therapy is not an option to quit smoking during gestation and lactation because it, like tobacco smoke, program the offspring to become more prone to various diseases later in life and to induce premature aging of organs.

In conclusion, although tobacco use decreases in developed countries, it increases in developing countries. A decrease in tobacco use is not accompanied by an immediate decrease in tobacco related diseases. The prevalence in these diseases only decrease after several decades. Parental smoking induces programming of the offspring resulting in the late onset of tobacco related diseases in the offspring even if they never smoked themselves. Many of the adverse effects associated with smoking are attributed to nicotine, the addictive substance in tobacco smoke. Thus nicotine replacement therapy is not advisable for especially pregnant and/or lactating mothers. Several studies indeed showed that maternal nicotine exposure also induce programming in the offspring which result in abnormal structure and function of organs. This is thought to be due to premature aging.
